# Vis/NIR Spectroscopy and Chemometrics for Non-Destructive Estimation of Chlorophyll Content in Different Plant Leaves

**DOI:** 10.3390/s25061673

**Published:** 2025-03-08

**Authors:** Qiang Huang, Meihua Yang, Liao Ouyang, Zimiao Wang, Jiayao Lin

**Affiliations:** 1 School of Materials and Environmental Engineering, Shenzhen Polytechnic University, Shenzhen 518055, China; ouyangl@szpu.edu.cn (L.O.); 230900373@mail.szpu.edu.cn (Z.W.); 230900372@mail.szpu.edu.cn (J.L.); 2 Department of Environmental Engineering, Yuzhang Normal University, Nanchang 330103, China; 0015862@zju.edu.cn; 3 Institute of Urban Ecology and Environmental Technology, Shenzhen Polytechnic University, Shenzhen 518055, China

**Keywords:** vis/NIR spectroscopy, chlorophyll content, MSD methods

## Abstract

**Highlights:**

**What are the main finding?**

- The MSD method serves as a tool for selecting a representative calibration dataset in leaves.
- The leaves’ sensitive bands were in the ranges of 500–640 nm and 740–1100 nm.
- Cubist achieved a higher determination coefficient than PLSR.
- Vis/NIR can be used for assessing drought’s impact on plants’ chlorophyll.

**What is the implication of the main finding?**

- MSD method can be useful for selecting a representative calibration dataset in leaves.
- Vis-NIR are the invaluable tool for assessing drought impacts on plant health and productivity.

**Abstract:**

Vegetation biochemical and biophysical variables, especially chlorophyll content, are pivotal indicators for assessing drought’s impact on plants. Chlorophyll, crucial for photosynthesis, ultimately influences crop productivity. This study evaluates the mean squared Euclidean distance (MSD) method, traditionally applied in soil analysis, for estimating chlorophyll content in five diverse leaf types across various months using visible/near-infrared (vis/NIR) spectral reflectance. The MSD method serves as a tool for selecting a representative calibration dataset. By integrating MSD with partial least squares regression (PLSR) and the Cubist model, we aim to accurately predict chlorophyll content, focusing on key spectral bands within the ranges of 500–640 nm and 740–1100 nm. In the validation dataset, PLSR achieved a high determination coefficient (R^2^) of 0.70 and a low mean bias error (MBE) of 0.04 mg g^−1^. The Cubist model performed even better, demonstrating an R^2^ of 0.77 and an exceptionally low MBE of 0.01 mg g^−1^. These results indicate that the MSD method serves as a tool for selecting a representative calibration dataset in leaves, and vis/NIR spectrometry combined with the MSD method is a promising alternative to traditional methods for quantifying chlorophyll content in various leaf types over various months. The technique is non-destructive, rapid, and consistent, making it an invaluable tool for assessing drought impacts on plant health and productivity.

## 1. Introduction

Influenced by global climate change, droughts have become more frequent and severe worldwide, posing a consistent threat to food security and agricultural production systems [1]. Additionally, drought conditions in many regions are exacerbated by changes in precipitation patterns, altered snow accumulation and melt regimes, and increased evapotranspiration, all of which contribute to climate change [2]. Field experiments have demonstrated that seasonal droughts impact vegetation differentially across geographical regions, seasons, and species [3]. These droughts significantly affect plant phenological processes, which in turn affect plant growth [4]. Furthermore, groundwater decline can lead to potential plant mortality [5]. Recently, research has focused on the direct effect of water availability on plants [6], revealing that the weakening of leaf photosynthesis due to drought is the primary reason for plant decline [7].

Chlorophyll plays a crucial role in photosynthesis by capturing light energy and converting it into chemical energy, which is then stored in the organic compounds that the plant synthesizes [8]. Leaf chlorophyll content is indeed a critical indicator of vegetation’s photosynthetic capacity and nutritional status as it exhibits a strong correlation with both the photosynthetic capacity and the developmental stage of the vegetation [9]. Studies have reported the effects of drought stress on chlorophyll fluorescence parameters, phytochemical content, and antioxidant activities [10]. Chlorophyll plays a pivotal role in assessing the intensity of photosynthesis and the nutritional status of vegetation [11]. Therefore, the rapid and accurate acquisition of spatiotemporal continuous chlorophyll data is of utmost importance for analyzing the impact of drought stress and its applications in agriculture [12]. Such data can help in understanding the response of vegetation to drought stress and developing strategies to mitigate its effects on agricultural productivity.

The traditional methods for determining chlorophyll content involve destructive sampling, where leaves are extracted and their chlorophyll content is measured based on light absorption [13]. While these methods provide accurate results, they are laborious, time-consuming, and involve the destruction of samples, leading to environmental pollution, which can be seen in the Table 1, from that we can see the advantages and disadvantages of commonly employed chlorophyll determination methods. These limitations make it difficult to meet the needs for rapid, non-destructive, and large-area monitoring. Hyperspectral remote sensing technology offers an alternative solution. Spectral signals are closely related to the biochemical and structural properties of plants, making it possible to monitor plant growth and nutritional content (such as chlorophyll and nitrogen content) without damaging the plant structure. In particular, the visible and near-infrared region (vis/NIR) provides high resolution, combined with high speed, simplicity, and low cost. This region enables a thorough understanding of the physiological responses of plants to drought stress [14].

Utilizing hyperspectral remote sensing within the visible/near infrared (vis/NIR) spectrum, researchers can rapidly and non-destructively assess chlorophyll content along with other critical plant parameters across extensive areas. This technology is pivotal in enhancing our comprehension of plant responses to drought stress and in formulating effective strategies to mitigate its adverse effects on agriculture and ecosystems. Vis/NIR spectroscopy has proven to be effective for assessing the chlorophyll status in the leaves of various species. Gamon and Surfus [15] pioneered the use of spectral reflectance to rapidly evaluate the chlorophyll, anthocyanin, and xanthophyll levels in intact sunflower leaves. Subsequent studies have demonstrated the capability of vis/NIR spectroscopy for non-destructive chlorophyll assessment in diverse plants such as rape [16], rice [17], wheat [18], and Toona sinensis [19]. The use of the spectroscopy technique for chlorophyll content calculation has been widely considered [20,21,22,23]. However, previous research has predominantly concentrated on the leaves of individual plants, with limited studies employing vis/NIR spectroscopy across multiple plants over sequential dates. Moreover, there is a notable absence of published studies that integrate reflectance measurements with multivariate data analysis to estimate chlorophyll content in various wild plants. The existing gap mainly involves that the accuracy of vis/NIR spectroscopy for chlorophyll determination depends on the quality of the calibration model, which requires careful selection and preparation of reference samples, especially for chlorophyll from plants from different regions, different plant species, and different growth conditions.

**Figure 1:**
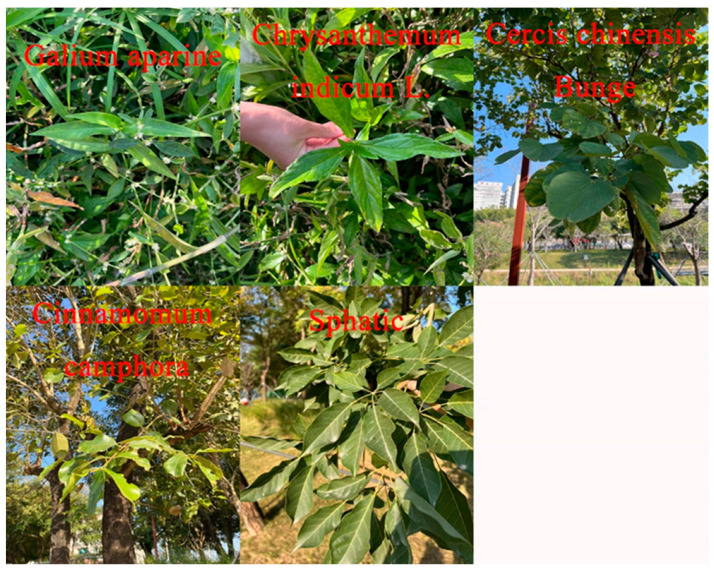
The leaves of the plants.

In our research, we selected five distinct plant species, each subjected to varying drought durations, as experimental subjects. We measured the visible to near-infrared (vis/NIR) spectra of several plant species, including *Spathiphyllum*, *Cinnamomum camphora*, *Chrysanthemum indicum*, *Galium aparine*, and *Cercis chinensis Bunge* (Figure 1), from February 2024 to November 2024 at monthly intervals. The objectives of our study were to (1) ascertain whether vis/NIR spectroscopy can accurately measure chlorophyll content in different plant leaves; (2) elucidate the underlying mechanisms of prediction; and (3) evaluate the representativeness of the samples selected for the calibration dataset, which is commonly used in in situ soil vis/NIR spectral analysis.

**Table 1:** The advantages and disadvantages of commonly employed chlorophyll determination methods.

Methods	The Main Processes	Advantages	Disadvantages
Spectrophotometer method [24]	Using the different solvents	Simple and quick	Long extraction time (24h) and solvents with high toxicity destroy the leaves
Fluorometry [25]	Sample preparation, solvent extraction, measurement, data Analysis	High sensitivity, quick	Cost, sensitivity to light conditions, using solvent
HPLC [26]	Pigment extraction, Mobile phase configuration, Chromatographic condition test, Drawing of working curves	High sensitivity, good Resolution	Heavy workload, complicated steps, long extracted time, or strict extraction conditions
Optoacoustic spectrometry [27]	Measure the amount of light absorbed by a sample	Simple and rapid inexpensive and easy to operate	Low accuracy, easily affected by temperature and light intensity
Vis/NIR spectroscopy	Spectral measurement, data analysis	Non-destructive, Rapid, portable, scalability	Depends on the quality of the calibration model

## 2. Materials and Methods

### 2.1. Leaf Sampling, Spectral Measurement, and Lab Chlorophyll Content Measurement

**Figure 2:**
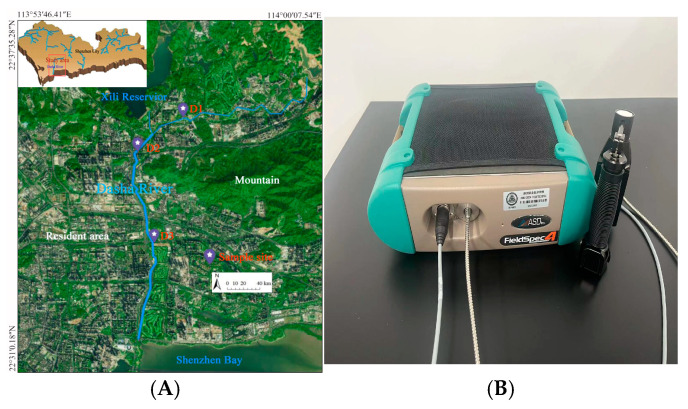
The location of sampling (**A**) and the spectra instrument (**B**).

The ecological restoration of the Shenzhen Dasha River Ecological Corridor, recognized as one of the top ten national spatial ecological restoration projects, was selected as the study area (Figure 2A). We collected samples from five plant species—*Spathiphyllum*, *Cinnamomum camphora*, *Chrysanthemum indicum*, *Galium aparine*, and *Cercis chinensis Bunge*—along the upstream, midstream, and downstream sections of the river. For each species, we collected seven replicate samples simultaneously from the same location over a period of ten consecutive months, yielding a total of 350 samples. Vis/NIR spectra of the leaves were measured using an ASD FieldSpec4 spectrometer, which operates across wavelengths from 350 to 2500 nm and is equipped with a fiber optic cable set at a 25° standard viewing angle. The sensor was positioned 150 cm above the ground, covering a detection area of approximately 0.4 m^2^ (Figure 2B). We converted the spectra to absorbance and used a Savitzky‒Golay smoothing filter with a first-order polynomial and window size of 15 to smooth the absorbance to diminish noise from the instrument and illumination. After measuring the spectra, the leaves were sent to the lab for chlorophyll content measurement.

Initially, we prepared the samples by weighing 0.5 g of leaves, which were then finely sliced into 1 mm wide filaments. These were subsequently placed in a test tube, sealed, and stored in darkness for a 24 h incubation period to facilitate extraction. Following this, 5 mL of a 1:1 solution, comprising equal parts acetone and absolute ethanol (5 mL each), was added to the test tube. Finally, we collected 1 mL of the resultant extract and combined it with 2 mL of a fresh mixture of acetone and absolute ethanol for further analysis.

After weighing the fresh leaves, they were placed into a mortar along with a measured quantity of calcium carbonate and quartz sand. Subsequently, an appropriate volume of either 80% acetone solution or 95% ethanol was added, and the mixture was thoroughly ground into a homogenate. This homogenate was then transferred to a centrifuge tube, and the mortar was rinsed with solvent to ensure complete extraction of chlorophyll. The contents of the centrifuge tube were centrifuged to separate the leaf fragments from the extract. The absorbance of the extract was measured using a spectrophotometer at wavelengths of 663 nm and 645 nm, corresponding to the maximum absorption peaks of chlorophyll a and chlorophyll b, respectively [28]. Further details can be found in the study by Gu et al. [29].

### 2.2. The Reprehensive Calibration Samples Selected

To investigate whether the mean squared Euclidean distance (MSD) method, previously utilized in soil spectra for selecting the optimal number of subset representations for calibration, is applicable to leaf spectra, we initially selected subsets ranging from 60 to 260 in increments of 40 from a total of 280 samples. These samples were chosen from an overall pool of 350 using the Kennard–Stone (KS) method [30]. For each subset i, Gaussian kernel density estimates of the probability density function (PDS) from the jth PC of vis/NIR of the leaves were calculated, which were called P_s_(*x_j_* ∈ ss). In addition, in validation dataset (n = 70), PDS from the *j*th PC of vis/NIR of leaves within the same bandwidth (a,b) and kernel as P_s_(*x_j_* ∈ ss), called P_p_(*x_j_*), were calculated too. Next, the MSD of P*_j_*(*x_j_* ∈ ss) and P_p_(*x_j_*) with k PCs were calculated.

MSD, used to select the optimal subset, was first proposed by Ramirez-Lopez et al. [31] in soil (Equations (1) and (2)) and revised by Yang et al. [32]:

(1) $$msd=\frac{1}{k}\sum_{j=1}^{k}d^{2}(P_{s}\left(x_{j}\in ss\right),P_{p}(x_{j}))$$

where

(2) $$d^{2}\left(P_{s}\left(x_{j}\in ss\right),P_{p}\left(x_{j}\right)\right)=\int_{a}^{b}{\left(P_{p}\left(x_{j}\right)-P_{s}\left(x_{j}\in ss\right)\right)}^{2}dx_{j}$$

Further, a and b represent the range of the *j*th PC, P_s_(x_j_ ∈ ss) and P_p_(x_j_) are the kernel density estimates of the *j*th PC at the range of a and b from each selected subset (i.e., 60, 100, 140, …280; the total *i* is 6), and, in the validation population (70), d^2^ means the squared Euclidean distance between the *i*th (*i* is in 1:6) subset and validation; k is the total number of PCs that are used, and MSD is the mean squared distance between two probability densities.

### 2.3. Models

Partial least squares regression (PLSR) is a multivariate statistical method employed to establish calibrations between reflectance spectra and crop variables, addressing collinearity issues [33]. Widely applied in soil spectroscopic research [34], a critical step in PLSR involves determining the optimum number of latent variables (LVs). This is achieved through a leave-one-out cross-validation procedure that minimizes the root mean square error.

Cubist is a rule-based predictive model that utilizes regression trees. The input space is segmented into various regions by the regression tree, and a simple linear model is applied to each region to generate predictions. The performance of the Cubist model is influenced by two hyperparameters: neighbors and committees, which are optimized through tuning. A nearest neighbor algorithm is employed at the leaf node to produce a prediction, which is then averaged with the results of a regression model at the same node to form the final output. The number of committees is adjusted to correct errors from previous models. In this study, a grid search involving committees c (2, 4, 6, and 8) and neighbors c (10, 20, 30, and 40) was conducted using a 10-fold cross-validation to determine the optimal settings. A more comprehensive and detailed description of the Cubist model is available in Henderson et al. [35].

We employed the coefficient of determination (R2), root mean squared error (RMSE), ratio of performance to interquartile distance (RPIQ), and bias to evaluate the performance of the vis/NIR spectra model across various modeling methods.

## 3. Results

### 3.1. Statistics for Sampling Information and Spectroscopy Analysis

To develop a spectral prediction model with high accuracy, appropriate samples from the calibration set were used. The statistical data from both the calibration and validation datasets, as well as a randomly selected subset from the calibration dataset and data from five different plant types, are presented in Table 2. The chlorophyll content range in the validation dataset fell within that of the calibration dataset, ensuring the robustness of the derived models. When the subset size was 220, the mean chlorophyll content (1.60 mg g^−1^) was closer to that of the calibration dataset (mean of 1.57 mg g^−1^) compared to the other subsets, indicating that these 220 samples are more likely to represent the entire calibration dataset effectively.

When examining the chlorophyll content across five plant species, significant differences were observed in the mean values. Specifically, *Galium aparine* exhibited the highest chlorophyll content, while *Cinnamomum camphora* had the lowest. Among the calibration dataset, *Galium aparine* and *Sphatic* displayed the highest and lowest chlorophyll contents, respectively.

**Figure 3:**
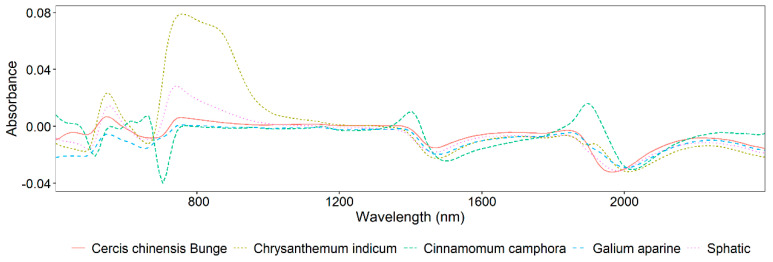
The reflectance of five plants.

The absorbance vis/NIR spectra of leaves from five plant species, smoothed using the Savitzky–Golay (SG) method, are depicted in Figure 3. This figure distinctly demonstrates that the original spectra of all the samples significantly differ across the wavelength range of 400–1200 nm. Notably, *Chrysanthemum indicum*, *Sphatic*, and *Cercis chinensis Bunge* exhibit pronounced peaks around 600 nm and 740 nm, as shown in Figure 3. Between 1200 nm and 2400 nm, the spectra are generally similar, with the exception of *Cinnamomum camphora*. Furthermore, there are notable absorbance peaks in the ranges of 1400–1500 nm and 1900–2000 nm.

### 3.2. The Optimal Number of Subset Samples in the PC Space of the Vis/NIR Data

**Figure 4:**
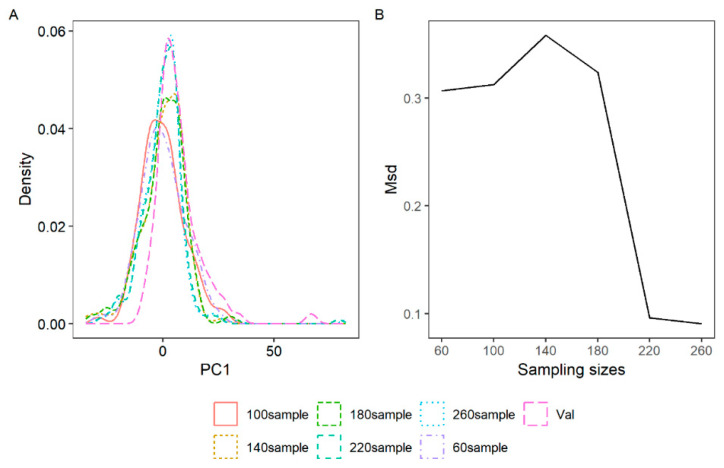
The density of the first six principal components of the vis/NIR leaf data in the subset (60, 100, …, 260) and the validation set (**A**), along with the mean squared Euclidean distance (MSD) between the probability density functions of the selected subset and the validation set, were analyzed (**B**).

The density distribution values of the first principal components (PCs) from randomly selected serial subset samples were significantly similar to those of the first PC of the validation set when the sample size exceeded 220 (Figure 4A). The mean squared Euclidean distance (MSD) between the probability density functions of the subset and validation samples initially increased as the subset size reached 140, then rapidly decreased upon reaching 220 samples, and subsequently declined more gradually beyond 260 samples (Figure 4B). The consistency in density distribution and MSD values shown in Figure 4 suggests that a subset of 220 samples optimally represents the validation samples while considering the labor and analytical costs (e.g., chlorophyll content via wet chemistry) from the calibration dataset. These findings may be beneficial for remote sensing monitoring as they eliminate the need for prior knowledge of chlorophyll content.

**Figure 5:**
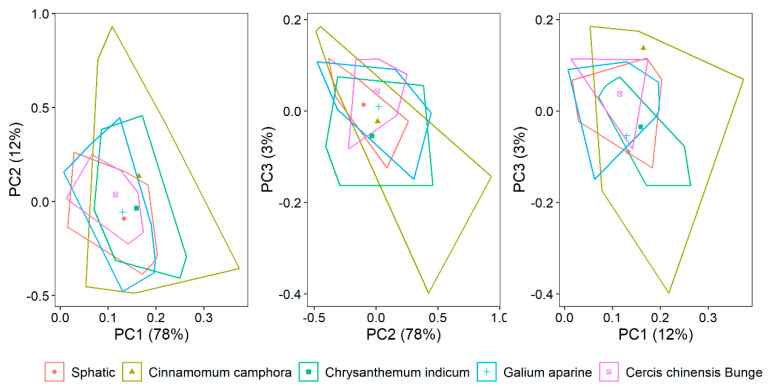
The convex hulls and their centroids plotted in the principal component (PC) space from five different plants.

Figure 5 displays the scores of the first three principal components derived from the leaves of five different plants. These three principal components account for 93% of the total variation, with the first component alone explaining 78%. Among the plants, *Cinnamomum camphora* covers a broader spectrum of space compared to the other four. The centroids of the first two principal components for all five plants are closely positioned, suggesting a narrow range of variation among them.

### 3.3. Model to Predict the Chlorophyll Content of Five Types of Plant Leaves

**Figure 6:**
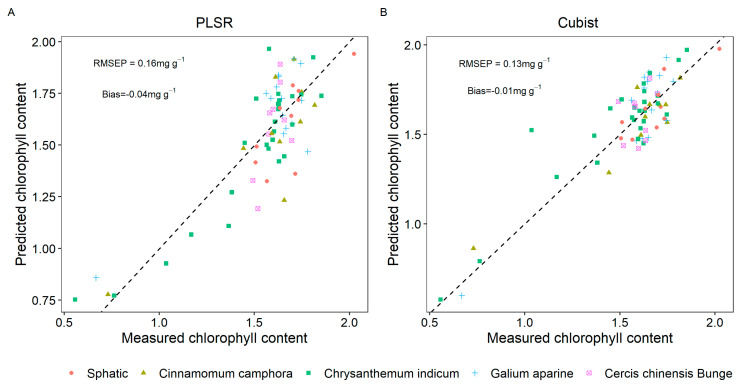
Scatterplots depict the relationship between measured and predicted chlorophyll content using two models: the partial least squares regression (PLSR) (**A**) and the Cubist model (**B**). The dashed line represents the 1:1 line.

The bias values for the chlorophyll prediction models of PLSR and Cubist ranged from −0.04 to −0.01, suggesting that both models achieved satisfactory fitting results. In the PLSR model, the predictions for chlorophyll content with lower measured values tended to be overestimated, while those with higher measured values were also somewhat overestimated. In comparison, the Cubist model provided superior predictions, with both the PMSEP and bias values (RMSEP = 0.13 mg g^−1^; bias = −0.01 mg g^−1^) being lower than those of the PLSR model (RMSEP = 0.16 mg g^−1^; bias = −0.03 mg g^−1^) (Figure 6). However, when analyzing predictions for chlorophyll content in plant leaves, there were no discernible patterns observed in either the PLSR or Cubist model.

**Figure 7:**
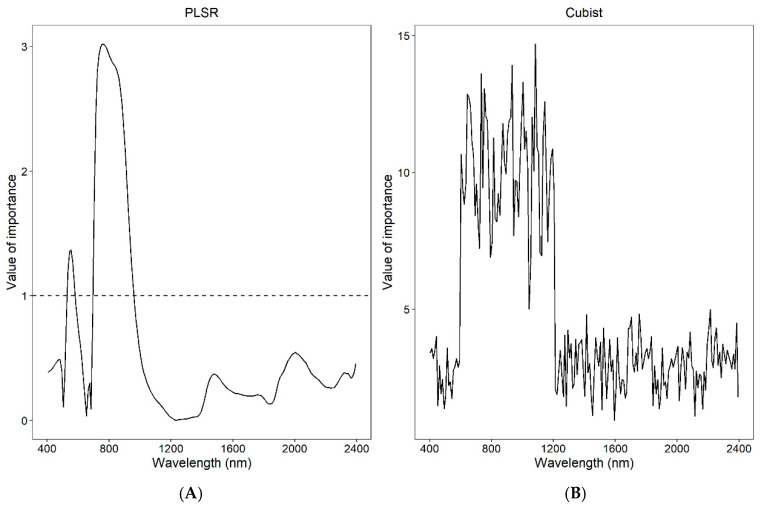
The coefficients of the partial least squares regression (PLSR) model (**A**) and the Cubist model (**B**) are depicted. In model A, the dashed line indicates wavelengths with values exceeding 1, highlighting their significance in the PLSR model.

In PLSR, the variable importance in projection (VIP) scores depicted in Figure 7 indicate that the critical wavelengths for predicting chlorophyll content are primarily within the ranges of 500–640 nm and 740–1100 nm, with peaks near 600 nm and 820 nm. Conversely, in the Cubist model, the VIP scores span a broader range from 600 nm to 1100 nm, and no distinct peaks are observed across the entire visible/near infrared (vis/NIR) spectrum.

## 4. Discussion

### 4.1. The Selected Representative Calibration Dataset

The consistency of the density distribution values from the first principal components (PCs) of randomly selected serial subset samples, along with the mean squared deviation (MSD) values, demonstrates the feasibility of representative calibration dataset methods when applied to plant leaves. Initially utilized in laboratory soil visible/near infrared (vis/NIR) spectra [36], the MSD approach was subsequently adapted for in situ vis/NIR spectra with modified algorithmic steps [32]. Despite the shift from soil to leaves, the method remains effective. This efficacy likely stems from the MSD method’s foundation in density distributions, a core aspect of multivariate statistical methods. This foundation enables the selection of samples that closely resemble those in the calibration dataset and, by extension, those in the validation dataset. The similarity among the five types of leaves in the vis/NIR spectra, also observable in PCA analysis, supports this. Although the leaves originated from different plants and were collected in various periods, the MSD method facilitates the selection of representative samples without the need for labor-intensive laboratory chemical measurements, thereby saving time and effort. However, this study was limited by the sampling locations and the fact that only five types of plants were examined. Broader application of this method requires further research across a more extensive range of plants and a larger geographical area.

### 4.2. The Principles of Prediction of Chlorophyll Content Using Vis/NIR Spectra

The RMSEP and bias values associated with the predicted results from the PLSR and Cubist models demonstrate that vis/NIR spectra can effectively predict chlorophyll content in various leaves. This finding aligns with the research conducted by Fernàndez-Martínez et al. [37], who illustrated that near-infrared reflectance spectroscopy facilitates the rapid and simultaneous evaluation of chloroplast pigments (chlorophylls a and b, lutein, neoxanthin, and beta-carotene) in Populus spp. leaves. Although their study reported a higher R^2^ value of 0.827 using PLSR compared to our study’s R^2^ of 0.70, this discrepancy may be attributed to the greater chlorophyll variance in our samples (SD = 0.32 g kg^−1^) as opposed to theirs (SD approximately 0.06 g kg^−1^).

Cubist models generally yield more accurate predictions than PLSR, demonstrating that nonlinear models can capture more information related to chlorophyll than linear models. This finding aligns with the research conducted by Rasooli Sharabiani et al. [18], which indicated that PLS modeling achieved more accurate predictions, with an R^2^ of 0.97, compared to PLSR, which also reported an R^2^ of 0.97. A key advantage of the Cubist model is its ability to utilize a broader range of VIP wavelengths, as illustrated in the VIP plot (Figure 7).

Plant pigments and phytonutrients are quantified using vis/NIR spectroscopy as these compounds exhibit characteristic chemical bonds that manifest as signal peaks in the spectral data, with the intensity of these peaks correlating to their concentrations [38]. Our study identified significant bands for chlorophyll content in the ranges of 500–640 nm and 740–1100 nm, aligning closely with the findings of Min and Lee [39], who identified critical wavelengths for chlorophyll detection at 448, 669, 719, 1377, 1773, and 2231 nm. Li et al. [40] noted strong chlorophyll absorption in the visible and near-infrared spectra at 1087, 1215, and 2219 nm. The variations in these results are primarily attributed to differences in plant water content as water in leaves exhibits distinct absorption bands at 760, 970, 1190, 1450, and 1940 nm [41]. Consequently, leaf water content can influence the depth of spectral absorption and affect the accuracy of predictive models. In this study, the prediction models for chlorophyll achieved an R^2^ of 0.70, indicating satisfactory model performance, although there is room for improvement. Future research should further explore the impact of factors such as moisture on model performance to enhance accuracy.

**Figure 8:**
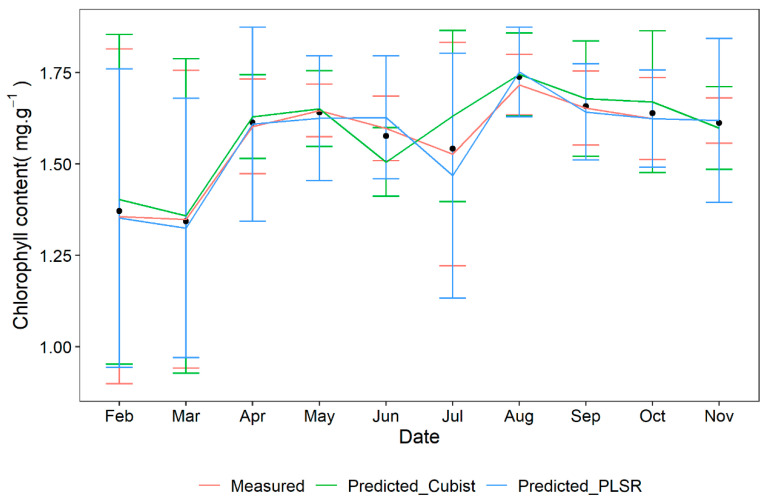
The variations in chlorophyll content across leaves from five different species were monitored over several months. The data presented are the averages of the outcomes measured by the PLSR and Cubist models. These predicted results for each treatment are expressed as the mean ± standard error (SE), the black point shows the mean value.

Chlorophyll content is a critical indicator for assessing plant responses to drought stress [42]. In our study, the measured chlorophyll content exhibited an increasing trend from February to August, peaking in August (Figure 8), consistent with the findings by Wang et al. [33], who also observed a peak in August followed by a gradual decrease. According to Shao et al. [43], August is the driest month in Shenzhen city, contributing to urban resilience under climate change. Therefore, the rise in chlorophyll content during this month may be attributable to several factors, including seasonal variations, temperature fluctuations, and plant physiological responses. Specifically, the increase might be linked to heightened photosynthetic activity during the growing season and favorable environmental conditions for chloroplast development in the leaves, alongside possible shifts in plant nutrient uptake [44]. Furthermore, both the PLSR and Cubist models predicted a chlorophyll content trend similar to the observed measurements across the months. Therefore, vis/NIR spectra can serve as an effective tool for monitoring the impact of drought stress on plants.

## 5. Conclusions

This study evaluated the method of selecting representatives using the mean squared deviation (MSD) value for calibration, in conjunction with partial least squares regression and Cubist techniques. Based on the results, we reached the following conclusions:

(1) The MSD method serves as a tool for selecting a representative calibration dataset in leaves.
(2) The sensitive bands of leaves were in the ranges of 500–640 nm and 740–1100 nm.
(3) Cubist achieved a higher determination coefficient than PLSR.
(4) Vis/NIR spectroscopy has proven to be effective in estimating chlorophyll content across five different types of leaves over various months.

This approach provides a portable, consistent, efficient, rapid, and non-destructive means of measuring chlorophyll levels. Furthermore, it is applicable to small leaf areas and can be utilized to monitor changes in chlorophyll content within the same plant over time, particularly in drought conditions. Future research should evaluate the ability of vis/NIR to predict chlorophyll content across a wider range of species and environmental conditions and investigate how environmental factors (e.g., light intensity, temperature, and soil moisture) and plant physiological status (e.g., nitrogen metabolism and stress responses) affect vis/NIR signatures, which could lead to the development of adjusted models that account for these variations.

## Figures and Tables

**Table 2:** Statistics of chlorophyll content of datasets of calibration and validation and the different plants.

Dataset	Max	Minimum	Mean	Stdev	Skew
Calibration (280)	3.03	0.79	1.57	0.46	0.23
Validation (70)	2.78	0.96	1.68	0.32	0.33
60	3.03	0.89	1.63	0.34	0.37
100	2.98	0.81	1.66	0.35	0.31
140	2.91	0.79	1.70	0.38	0.34
180	3.03	0.88	1.69	0.41	0.23
220	3.01	0.81	1.60	0.44	0.24
*Galium aparine* (70)	2.11	0.96	1.47	0.32	0.33
*Chrysanthemum indicum* (70)	3.03	1.33	1.95	0.63	0.68
*Cercis chinensis Bunge* (70)	2.01	1.74	1.88	0.14	−0.02
*Cinnamomum camphora* (70)	1.45	0.79	1.10	0.33	0.14
*Sphatic* (70)	1.84	1.67	1.77	0.09	−0.34

## Data Availability

Data will be made available on request.
